# Prevalence and Risk Factors of Postoperative Spinal Infections: A Five-year Study in Northwestern Iran

**DOI:** 10.34172/aim.34624

**Published:** 2025-09-01

**Authors:** Seyed Taher Mousavi, Farhad Mirzaei, Mohammad Shimia, Moslem Shakeri

**Affiliations:** ^1^Department of Neurosurgery, Faculty of Medicine, Tabriz University of Medical Sciences, Tabriz, Iran

**Keywords:** Microorganism, Postoperative infections, Risk factors, Spine, Surgery

## Abstract

**Background:**

Surgical site infections (SSIs), particularly after spinal procedures, remain a major concern despite advances in infection control. This study aimed to determine the prevalence and associated risk factors of postoperative spinal infections in northwestern Iran.

**Methods:**

A cross-sectional study was conducted on 500 adult patients (≥18 years) who underwent spinal surgery in two referral hospitals in Tabriz (Imam Reza and Shohada) between March 2019 and February 2024. Postoperative infections were confirmed by infectious disease specialists. Data on demographics and surgical variables (including surgical site, approach, duration, blood loss, and transfusion) were collected using a standardized checklist. Statistical analyses were conducted in SPSS version 23.0 using Chi-squared and independent samples t-tests. Logistic regression was performed to estimate adjusted odds ratios (ORs) with 95% confidence intervals (CIs).

**Results:**

The prevalence of postoperative spinal infections was 6%. *Staphylococcus aureus* was the most common pathogen (66.7%). Significant risk factors included female sex, older age, corticosteroid use, diabetes mellitus, longer surgery duration (>4 hours), blood loss (>1 liter), and blood transfusion (*P*<0.05). Diabetes (OR=5.90, 95% CI: 2.30–15.20) and prolonged surgery (OR=6.90, 95% CI: 2.50–19.00) showed the strongest associations. No significant associations were found for BMI, smoking, hypertension, CRP, ESR, surgical site, or technique.

**Conclusion:**

A 6% infection rate was identified, with several clinical and demographic factors increasing risk. Recognizing these predictors is essential for prevention. Broader, multi-center studies are recommended to validate findings and inform national surgical infection control policies.

## Introduction

 Despite significant advances in medical care, particularly in surgical techniques, managing postoperative infectious complications remains one of the major challenges in healthcare systems.^1^ Surgical site infections (SSIs) are the most prevalent infectious complication in surgical wards and the second most frequent type of hospital-acquired infection, accounting for approximately 25% of all hospital infections.^2^ The occurrence of these infections could lead to treatment failure, prolonged hospitalization, increased healthcare costs and even mortality.^3^

 Previous studies have demonstrated that numerous factors contribute to the development of SSIs, which could be broadly categorized into patient- and surgery-related factors.^4^ The first category includes host-related factors, which are often non-modifiable or difficult to control. These factors include old age, obesity, diabetes, smoking, previous surgeries or infections at the same site, previous radiation therapy, chronic skin conditions such as psoriasis, immune and nutritional status and adherence to postoperative care.^5^ In the second category, surgical factors, such as type of surgery (e.g. posterior approach), placement of metallic implants, bone graft harvesting, excessive bleeding, duration of surgery, adherence to sterile techniques, traffic in the operating room, instrument contamination and use of intraoperative fluoroscopy, are key factors that contribute to the development of infections. Moreover, the rising prevalence of resistant pathogens such as methicillin-resistant *Staphylococcus aureus* (MRSA) and gram-negative bacteria has further complicated this challenge.^6^

 Among various types of surgeries, spinal surgeries are at higher risk for SSIs due to their complexity, prolonged operative duration and patients’ specific conditions (such as old age, chronic diseases, or use of immunosuppressive medications).^7,8^ SSIs are considered the third most prevalent complication following spinal surgeries.^9,10^ These infections result in patient readmission, adverse outcomes and substantial additional costs. In the United States, this issue results in an estimated $1 to $10 billion in direct and indirect medical costs annually and is associated with approximately 8,000 deaths per year.^11^ Additional risk factors for infection in these patients include the use of drains and catheters, hospitalization for more than 48 h, revision surgeries and the need for blood transfusions. Some studies have reported postoperative infection rates of up to 12% following spinal surgery, which is considerably higher than those observed after other types of surgical procedures.^12,13^ However, despite this broad body of literature, there is considerable variability in the methods, populations, and definitions used across studies, making comparisons and generalizations difficult.^14,15^ Additionally, many studies fail to quantify the relative impact of individual risk factors or control for confounding variables, which limits their practical applicability.^16^ These limitations highlight the need for more structured and context-specific evaluations.

 In addition, most existing literature comes from high-income countries, and there is a noticeable lack of regional data from the Middle East and North Africa (MENA) region, including Iran. Despite the clinical importance of SSIs in these settings, epidemiological studies specifically focusing on spinal surgeries remain scarce, fragmented, or outdated.^17^ Moreover, previous studies often remain descriptive and do not offer critical analysis regarding the relative impact or interaction of various risk factors. This lack of analytical perspective makes it difficult to prioritize interventions or adapt global guidelines to local contexts.

 Even with the implementation of preventive strategies (such as prophylactic antibiotics, strict sterilization protocols, and staff training) a substantial number of postoperative spinal infections continue to occur. In low- and middle-income countries, one major reason for this is the limited availability of reliable data regarding both the prevalence and the specific risk factors relevant to local clinical settings.^17^ Lack of accurate data regarding the prevalence and relative contribution of each risk factor seems to be among the main reasons for the insufficient control of these complications.^18^ Documenting the prevalence of postoperative spinal infections is of particular importance for several reasons, including preoperative consultation with the patient, improving the quality of services and, in some cases, addressing legal issues.^19-21^

 Therefore, to address this gap in both regional epidemiology and analytical evaluation of risk factors, the present study was conducted to investigate the prevalence of postoperative infections following spinal surgeries and identify the associated risk factors in two major teaching and treatment centers in Tabriz, namely Imam Reza and Shohada hospitals, which serve as specialized and subspecialized referral centers in northwestern Iran.

## Materials and Methods

###  Study Design, Setting and Sampling

 This cross-sectional study was conducted on the medical records of patients undergoing spinal surgeries in two specialized referral centers, Imam Reza and Shohada hospitals, in Tabriz, northwestern Iran, from March 2019 to February 2024. The minimum sample size was estimated to be 100 patients with postoperative infections using Cochran’s formula, considering α = 0.05, absolute error (d) of 0.1 and *P* = 0.5. However, all eligible medical records within the study period were included using a census sampling method, resulting in a total sample size of 500 patients. This approach was chosen to enhance the statistical power and generalizability of the findings. Inclusion criteria were patients over 18 years of age who had undergone spinal surgery, availability of clinical documentation, including laboratory results (CBC, ESR and CRP), pre- and postoperative radiographic images, a detailed surgical report in the medical records and documented pre- and postoperative clinical examinations. Records with missing essential data (such as laboratory results or surgical reports) were excluded from the study.

 After obtaining ethical approval and access to the hospitals’ medical archives, the researcher (a trained resident) systematically reviewed and identified all spinal surgery records. Data were collected through manual review of both electronic and paper-based medical records, as the hospital archives contained a mix of structured electronic data and unstructured paper files. In the initial phase, records containing diagnoses and surgical reports were extracted. Subsequently, cases with a confirmed diagnosis of SSIs, verified by an infectious disease specialist, were identified. The diagnosis of SSIs was based on clinical signs and symptoms, supported by microbiological confirmation through laboratory cultures when available, and validated by an infectious disease specialist to ensure diagnostic accuracy. Patient data were recorded in a standard data collection checklist and later reviewed by the research team to enhance accuracy. To reduce potential information bias, data extraction was independently performed by two researchers and discrepancies were resolved through discussion and review by a third investigator.

 The checklist consisted of two main sections. The first section included demographic and background information such as gender, age, history of diabetes and hypertension, body mass index (BMI), smoking status, use of immunosuppressive medications, history of previous surgeries (none, one, or multiple), history of previous SSIs, and preoperative white blood cell (WBC) count and C-reactive protein (CRP) levels.

 The second section covered surgical details including the surgical site (cervical, thoracic, lumbar, or sacral), type of surgery (discectomy, decompression, fusion, osteotomy, or debridement), surgical duration (categorized as less than 2 hours, 2–4 hours, or more than 4 hours, with “longer surgery duration” defined as more than 4 hours), blood loss volume (defined as less than or more than 1 liter), and number of blood transfusion units (categorized as none, one unit, two units, or more than two units). The timing of blood transfusion was not included in the present analysis.

 This observational study was reported in accordance with the STROBE (Strengthening the Reporting of Observational Studies in Epidemiology) checklist to ensure transparency and completeness in reporting.

###  Statistical Analysis

 The data were analyzed using SPSS 23.0. The normality of quantitative variables was first assessed using Kolmogorov-Smirnov test. For the description of quantitative variables (if normally distributed), mean and standard deviation were used, while frequency and percentage were used for qualitative variables. The correlation between qualitative variables and occurrence of infection was examined using the Chi-squared test. The difference in quantitative variables between the two groups (infected and non-infected) was assessed using the independent samples *t*-test. Additionally, to identify independent risk factors associated with postoperative spinal infections, a multivariate logistic regression analysis was performed, reporting adjusted odds ratios (ORs) with 95% confidence intervals (CIs) and the corresponding *P *values. A *P *value of less than 0.05 was considered statistically significant.

## Results

###  Demographic and Background Characteristics of the Study Sample

 As shown in Table 1, the mean age of the patients was 42.78 years ( ± 5.5), with an approximate age range of 26 to 59 years, and their mean weight was 82.18 kg ( ± 6.5). In terms of gender, 69% were female and the remaining were male. Smoking was reported in 15.6% of the patients, history of previous surgeries in 10%, history of corticosteroid use in 17.2%, diabetes mellitus in 14% and hypertension in 24.8%.

**Table 1:** Demographic and Background Characteristics of the Studied Patients (*n* = 500)

**Qualitative variables**	**Variable levels**	**Frequency (%)**
Gender	Male	155 (31.0)
	Female	345 (69.0)
Smoking	No	422 (84.4)
	Yes	78 (15.6)
History of surgery	No	450 (90.0)
	Yes	50 (10.0)
History of corticosteroid use	No	414 (82.8)
	Yes	86 (17.2)
History of hypertension	No	376 (75.2)
	Yes	124 (24.8)
History of diabetes	No	430 (86.0)
	Yes	70 (14.0)
**Quantitative variables**		**Mean (SD)**
Age (years)		42.78 (5.5)
Weight (kg)		82.18 (6.5)

###  Surgical and Therapeutic Characteristics of the Study Sample

 In this study, the mean (standard deviation) levels of preoperative inflammatory markers, including CRP and ESR, were reported at 10.78 ( ± 2.1) and 10.16 ( ± 2.5), respectively. The lumbosacral region (75%) was the most common surgical site, and the posterior approach (85%) was the most frequently applied approach. A combination of fusion and laminectomy (45%) was the predominant surgical method among the participants. Surgical duration lasted 2‒4 h in half of the patients (50%). Blood loss was less than 600 mL in more than half of the patients (62%). Additionally, 65% of the patients did not require blood transfusions. Further details on surgical and therapeutic characteristics are presented in Table 2.

**Table 2:** Surgical and Therapeutic Variables of the Studied Patients (*n* = 500)

**Qualitative variables**	**Variable levels**	**Frequency (%)**
Surgical site	Lumbosacral	375 (75.0)
	Cervical	100 (20.0)
	Thoracic	25 (5.0)
Approach	Posterior	425 (85.0)
	Anterior	65 (13.0)
	Anteroposterior	10 (2.0)
Surgical method	Laminectomy	150 (30.0)
	Fusion and laminectomy	225 (45.0)
	Laminectomy and diskectomy	125 (25.0)
Surgical duration	Less than 2 h	150 (30.0)
	2‒4 h	250 (50.0)
	More than 4 h	100 (20.0)
Blood loss volume	Less than 600 cc	310 (62.0)
	Between 600 cc and 1 liter	125 (25.0)
	More than 1 liter	65 (13.0)
Blood transfusion	None	325 (65.0)
	1 unit	115 (23.0)
	2 units	60 (12.0)
**Quantitative variables**		**Mean (SD)**
CRP		10.78 (2.1)
ESR		10.16 (2.5)

###  Prevalence of Infections Following Spinal Surgeries in the Study Sample

**Figure 1:**
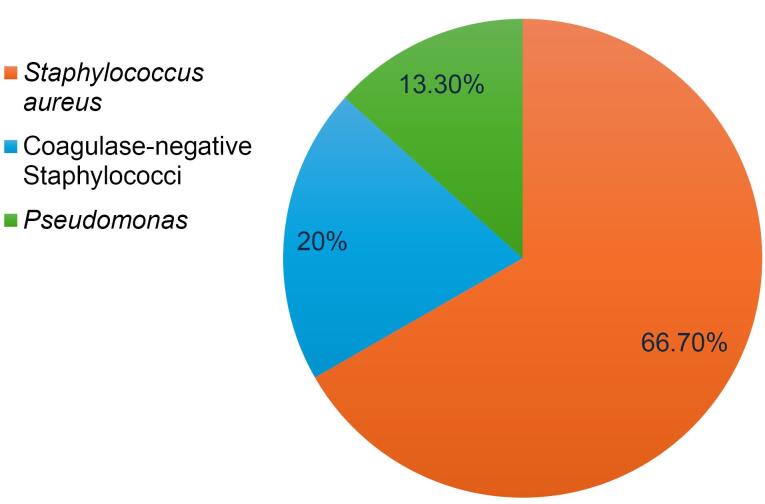
Distribution of Microorganisms in Postoperative Spinal Infections (*n* = 30)

 Overall, 30 patients (6% of the total sample) developed SSIs following spinal surgeries. *Staphylococcus aureus* was the most common infectious agent among the participants, followed by coagulase-negative staphylococci and *Pseudomonas* (Figure 1).

###  Comparison of Demographic and Background Variables Between the Two Patient Groups

Table 3 compares the demographic and background variables between infected and non-infected groups. The infection rate among females was 6.96%, compared to 3.87% among males (*P* = 0.021). Patients with a history of corticosteroid use had an infection rate of 26.74%, versus 1.69% in those without (*P* = 0.042). Similarly, the infection rate was 35.71% among diabetics compared to 1.16% in non-diabetics (*P* = 0.002). No significant differences were observed regarding weight, smoking status, history of previous surgeries, or hypertension (*P* > 0.05).

**Table 3:** Comparison of demographic and background variables between the two groups of studied patients (*n* = 500)

**Variables**	**Variable levels**	**Infection cases (** ***n*** **)**	**Non-infection cases (** ***n*** **)**	**Total cases (** ***n*** **)**	**Infection within group (%)**	***P *** **value**
Gender	Male	6	149	155	3.87	0.021*
	Female	24	321	345	6.96	
Smoking	No	12	410	422	2.84	0.230*
	Yes	18	60	78	23.08	
History of surgery	No	13	437	450	2.89	0.390*
	Yes	17	33	50	34.00	
History of corticosteroid use	No	7	407	414	1.69	0.042*
	Yes	23	63	86	26.74	
History of hypertension	No	15	361	376	3.99	0.190*
	Yes	15	109	124	12.10	
History of diabetes	No	5	425	430	1.16	0.002*
	Yes	25	45	70	35.71	
**Quantitative variables**		**Mean (SD)**		**Mean (SD)**		**Significance level**
Age		55.18 (6.8)		34.29 (2.5)		<0.001**
Weight		83.22 (8.2)		78.38 (4.5)		0.680**

* *P *value by chi-squared test, ** *P *value by independent samples *t*-test. significant *P* values are bold (values under 0.05).

###  Comparison of Surgical and Therapeutic Variables Between the Two Patient Groups

Table 4 presents the comparison of the surgical and therapeutic variables between patients with and without postoperative infections. As shown in the table, a surgical duration of more than 4 hours (with 20.0% infection rate in this group, *P* = 0.022), blood loss volume exceeding 1 liter (27.69% infection rate, *P* = 0.009), and receiving two units of blood transfusion (30.0% infection rate, *P* = 0.040) were significantly associated with the occurrence of SSIs. However, no significant correlation was found between postoperative infections and preoperative CRP and ESR levels, surgical site, surgical approach, or surgical method (*P* > 0.05).

**Table 4:** Comparison of Surgical and Therapeutic Variables Between the Two Groups of Studied Patients (*n* = 500)

**Variables**	**Variable levels**	**Infection cases (** ***n*** **)**	**Non-infection cases (** ***n*** **)**	**Total cases (** ***n*** **)**	**Infection within group (%)**	***P *** **value**
Surgical site	Lumbosacral	13	362	375	3.47	0.125*
	Cervical	12	88	100	12.0	
	Thoracic	5	20	25	20.0	
Approach	Posterior	14	411	425	3.29	0.361*
	Anterior	14	51	65	21.54	
	Anteroposterior	2	8	10	20.0	
Surgical method	Laminectomy	6	144	150	4.0	0.411*
	Fusion and laminectomy	12	213	225	5.33	
	Laminectomy and diskectomy	12	113	125	9.6	
Surgical duration	Less than 2 h	5	145	150	3.33	0.022*
	2‒4 h	5	250	255	1.96	
	More than 4 h	20	80	100	20.0	
Blood loss volume	Less than 600 cc	6	304	310	1.94	0.009*
	Between 600 cc and 1 liter	6	119	125	4.8	
	More than 1 liter	18	47	65	27.69	
Blood transfusion	None	5	320	325	1.54	0.040*
	1 unit	7	108	115	6.07	
	2 units	18	42	60	30.0	
**Quantitative variables**		**Mean (SD)**		**Mean (SD)**		**Significance level**
CRP		9.73 (4.1)		10.21 (2.0)		**0.528
ESR		11.16 (3.5)		10.01 (2.1)		**0.633

* *P *value by chi-squared test, ** *P *value by independent samples *t*-test. significant *P* values are bold (values under 0.05).

Table 5 presents the results of the multivariate logistic regression analysis evaluating independent risk factors associated with postoperative spinal infections. Adjusted ORs with their corresponding 95% CIs and *P*-values are reported for each category of the studied variables, with the reference groups clearly indicated. The analysis revealed that smoking significantly increases the risk of infection, with an adjusted OR of 3.80 (95% CI: 1.60 to 8.90, *P* = 0.003). A history of previous surgery and corticosteroid use were also significant risk factors, with adjusted ORs of 3.00 (95% CI: 1.20 to 7.50, *P* = 0.020) and 4.20 (95% CI: 1.70 to 10.30, *P* = 0.002), respectively. Diabetes mellitus demonstrated the strongest association with postoperative infection, with an adjusted OR of 5.90 (95% CI: 2.30 to 15.20, *P* < 0.001). Surgical site played an important role as well, with surgeries performed in the cervical and thoracic regions associated with higher infection risks compared to the lumbosacral region (adjusted ORs 3.70 and 6.80, respectively). Furthermore, surgical duration exceeding four hours and blood loss greater than one liter were strongly linked to increased infection risk, with adjusted ORs of 6.90 (*P* < 0.001) and 18.50 (*P* < 0.001), respectively. Blood transfusion was also a significant factor, with receiving one or two units increasing the infection risk (adjusted ORs of 3.90 and 25.50, respectively). Other variables such as male gender, history of hypertension, and surgical methods showed elevated ORs but did not reach statistical significance. These findings emphasize the importance of managing comorbidities like diabetes and corticosteroid use, along with optimizing surgical factors such as minimizing operative time and blood loss, to reduce the likelihood of postoperative spinal infections.

**Table 5:** Multivariate Logistic Regression Analysis of Risk Factors for Postoperative Spinal Infections

**Variable**	**Level**	**Postoperative spinal infections**	
		**Adjusted OR (95% CI)**	***P*** ** value**
Gender	Male	1.50 (0.70 – 3.20)	0.30
	Female	Ref	Ref
Smoking	Yes	3.80 (1.60 – 8.90)	0.003
	No	Ref	Ref
History of surgery	Yes	3.00 (1.20 – 7.50)	0.020
	No	Ref	Ref
History of corticosteroid use	Yes	4.20 (1.70 – 10.30)	0.002
	No	Ref	Ref
History of hypertension	Yes	2.10 (0.90 – 4.80)	0.080
	No	Ref	Ref
History of diabetes	Yes	5.90 (2.30 – 15.20)	< 0.001
	No	Ref	Ref
Surgical site	Cervical	3.70 (1.40 – 9.70)	0.008
	Thoracic	6.80 (2.20 – 21.30)	0.001
	Lumbosacral	Ref	Ref
Approach	Anterior	7.40 (2.90 – 18.70)	< 0.001
	Anteroposterior	6.80 (1.30 – 35.40)	0.022
	Posterior	Ref	Ref
Surgical method	Fusion and laminectomy	2.10 (0.80 – 5.70)	0.130
	Laminectomy and diskectomy	2.40 (0.90 – 6.60)	0.086
	Laminectomy	Ref	Ref
Surgical duration	2‒4 h	0.60 (0.18 – 2.00)	0.41
	> 4 h	6.90 (2.50 – 19.00)	< 0.001
	< 2 h	Ref	Ref
Blood loss volume	600 cc - 1 liter	2.30 (0.80 – 6.80)	0.11
	> 1 liter	18.50 (7.00 – 48.80)	< 0.001
	< 600 cc	Ref	Ref
Blood transfusion	1 unit	3.90 (1.20 – 12.30)	0.024
	2 units	25.50 (8.60 – 75.60)	< 0.001
	None	Ref	Ref

## Discussion

 The present study was conducted to investigate the prevalence of postoperative infections following spinal surgeries and the contributing factors. The results indicated postoperative infections occurred in 30 cases (6%). These findings were consistent with those of numerous similar national and international studies, suggesting that the prevalence of SSIs following spinal surgeries is similar across different countries and healthcare centers, despite some differences in infection rates and pathogen types. For example, Zarei et al reported a relatively low infection rate (6.8% in the case group and 4.5% in the control group) from Al-Zahra Hospital in Isfahan.^22^ In the study by Nota et al on 5,761 patients, the incidence of postoperative infections within 90 days was reported at 6%.^23^ These alignments suggest homogeneity in the factors contributing to infection rates. However, some studies have reported differing results, which may be attributed to factors such as type of surgeries, geographical location, sample size and patient inclusion criteria. For example, in the study by Al-Gamdi et al in Saudi Arabia, the infection rate was 4%.^24^ In the studies by Liu et al in the United States^25^ and Abolfotouh et al in Africa,^17^ the infection rates were 2.4% and 4.2%, respectively. These observed differences underscore the heterogeneity across healthcare systems, which may reflect variations in surgical infrastructure, ICU admission policies, antibiotic stewardship, and infection control protocols. Without accounting for these contextual differences, direct comparisons between settings may be limited in interpretability.

 In the present study, among the common microbial factors, *S. aureus*, coagulase-negative staphylococci and *Pseudomonas* were identified as the most common pathogens associated with infections, respectively. In contrast, the study by Fakour et al in Razi hospital in Ahvaz reported *Pseudomonas* as the most common causative agent of infection, likely due to differences in settings and types of surgeries performed.^26^ Durkin et al found that the most common pathogens included *S. aureus*, coagulase-negative staphylococci and *Escherichia coli.*^27^ These differences may be attributed to variations in environmental conditions, study designs, populations studied and care practices at different centers, all of which could influence the final results. Such microbial variations could be also influenced by local antimicrobial policies, availability of infection surveillance systems, and differences in surgical asepsis standards, which were not consistently reported across studies.

 Importantly, this study went beyond descriptive statistics by presenting adjusted ORs for several independent risk factors. Among patient-related variables, diabetes mellitus emerged as the strongest risk factor for postoperative spinal infections (adjusted OR = 5.90; 95% CI: 2.30–15.20; *P* < 0.001). Corticosteroid use was also significantly associated with infection (adjusted OR = 4.20; 95% CI: 1.70–10.30; *P* = 0.002), as was smoking (adjusted OR = 3.80; 95% CI: 1.60–8.90; *P* = 0.003), and prior surgery (adjusted OR = 3.00; 95% CI: 1.20–7.50; *P* = 0.020). In terms of surgical factors, longer surgical duration ( > 4 hours) showed a strong association with infection (adjusted OR = 6.90; *P* < 0.001), as did blood loss exceeding 1 liter (adjusted OR = 18.50; *P* < 0.001). Blood transfusion further increased the risk significantly, especially in patients receiving two units (adjusted OR = 25.50; *P* < 0.001). Surgical site was another determinant: surgeries in the thoracic (adjusted OR = 6.80) and cervical (adjusted OR = 3.70) regions had higher risks compared to the lumbosacral region. The strength of these associations highlights the importance of risk stratification, but also points to the need for further studies that control for setting-specific practices, such as surgical team experience, infection prevention protocols, and postoperative ICU care.

 Consistent with our findings, Najafizadeh et al identified advanced age and a history of chemotherapy or radiotherapy as factors contributing to the occurrence of infection.^28^ However, in contrast to our results, no significant correlation was observed between infection and variables such as gender, smoking and diabetes. Furthermore, our findings were in line with other research that has confirmed the role of diabetes as a contributing factor to infections. Karamouzian et al identified diabetes as the most important risk factor for deep wound infections.^29^ Also, Soroush et al reported factors such as surgical duration, presence of drains, steroid use and low hemoglobin levels as independent predictors of infection.^30^ Mosleh et al reported a higher prevalence of infection among male patients and those under 35 years of age, particularly in emergency surgeries.^31^ Hojjat et al found a significant correlation between surgical wound infection and factors such as type of fracture, smoking, surgical duration, type of surgery and length of hospital stay.^32^ Additionally, Al-Gamdi et al showed that factors such as hypertension, prolonged hospital stay, prolonged surgical duration and use of multiple blood units play a significant role in increasing the risk of infection.^24^ Furthermore, Abolfotouh et al identified factors such as diabetes, smoking, reconstructive surgery and prolonged hospital stay as significant contributors to the occurrence of postoperative infections.^17^ Dong et al reported advanced age, prolonged surgical duration, blood loss exceeding 1,000 mL and history of diabetes as factors associated with an increased risk of SSIs.^33^ Similarly, Liu et al identified factors such as diabetes, low albumin and calcium levels, prolonged surgical duration, increased blood loss and decreased hemoglobin as major predictors of SSIs.^25^ These comparative findings, while valuable, should be interpreted cautiously due to likely heterogeneity in patient case-mix, surgical subspecialties, and hospital-level infection monitoring protocols that may not have been standardized across studies.

 Finally, it should be noted that the observed differences in findings may be attributed to several factors, including geographical location, sample size, type of surgeries, patients’ demographic characteristics, and differences in inclusion and exclusion criteria across studies. These differences could account for the discrepancy between our results and those of other studies.

 The results of this study, along with previous evidence, emphasize the importance of identifying high-risk patients before the surgery, closely monitoring patients during the procedure and ensuring the control of patient safety conditions. Using infection control protocols, reducing surgical duration and optimizing patient conditions before the surgery could help reduce the incidence of infections. Despite its significance, the present study has several limitations. First, its retrospective and cross-sectional design only allows for identification of associations, limiting causal inference and introducing potential biases inherent to retrospective data collection. Second, the study was conducted in a single region in northwestern Iran, which may affect the generalizability of the findings to other geographical areas or populations. Third, the microbial resistance profiles of isolated pathogens were not assessed, which could have provided important insights into treatment challenges. Finally, clinical outcomes related to postoperative infections, such as length of hospital stay or functional recovery, were not reported. Future research should aim to include multi-center data from diverse healthcare systems and explicitly examine how structural variations (such as ICU protocols, preoperative optimization strategies, and antibiotic stewardship) may affect infection rates. Prospective longitudinal design, broader geographic coverage, and inclusion of antimicrobial resistance and clinical outcomes are recommended.

## Conclusion

 In this study, the prevalence of postoperative spinal infections was reported at 6%. The most common microbial factors associated with infections were *S. aureus*, coagulase-negative staphylococci and *Pseudomonas*, in decreasing order of frequency. Data analysis revealed that certain variables, such as female gender, history of corticosteroid use, diabetes mellitus, surgical duration exceeding 2 hours, blood loss greater than 1 liter and receiving at least 2 units of blood transfusion, were significantly associated with an increased risk of postoperative infections. Additionally, the mean age of patients with infections was significantly higher than that of patients without infections. However, no significant correlation was found between the occurrence of infection and other variables, including weight, smoking, surgical history, hypertension, CRP, ESR, surgical site, type of approach or surgical method. Conducting further studies in other regions of the country could help identify factors influencing postoperative infections, develop preventive strategies and improve the quality of healthcare services in this field.
